# Supplementary material for: Phenotypic response of yeast metabolic network to availability of proteinogenic amino acids
Source: Front Mol Biosci. 2022 Aug 22;9:963548. doi: 10.3389/fmolb.2022.963548 (PMC9441596; doi:10.3389/fmolb.2022.963548)
Supplement: Supplementary file 1 [file DataSheet1.PDF]

Supplementary Material

1 SUPPLEMENTARY TABLES AND FIGURES

1.1 Figures

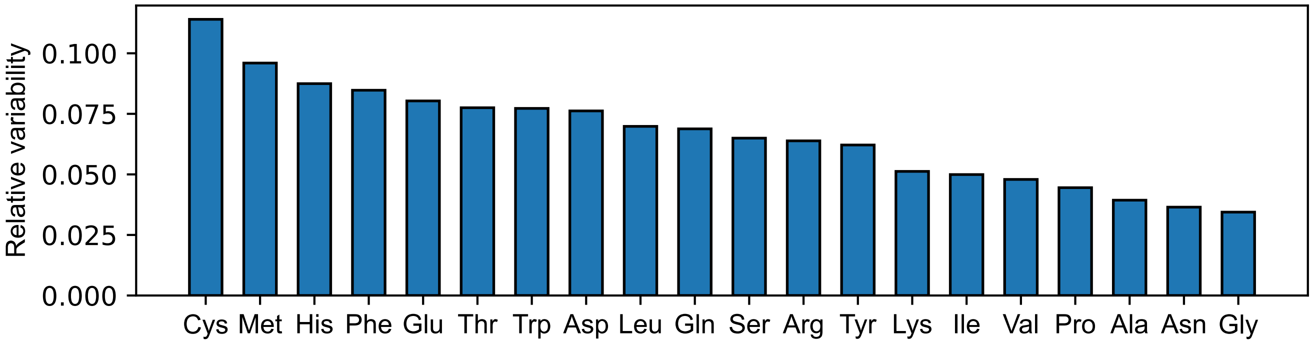

Figure S1. Rank-ordered, relative flux variability of proteinogenic amino acids on a defined, minimal glucose medium. Calculated by normalizing the feasible flux ranges at 99% of optimal growth by their corresponding mean flux value. Mean-flux normalized counterpart to the error bars in Fig. 1.

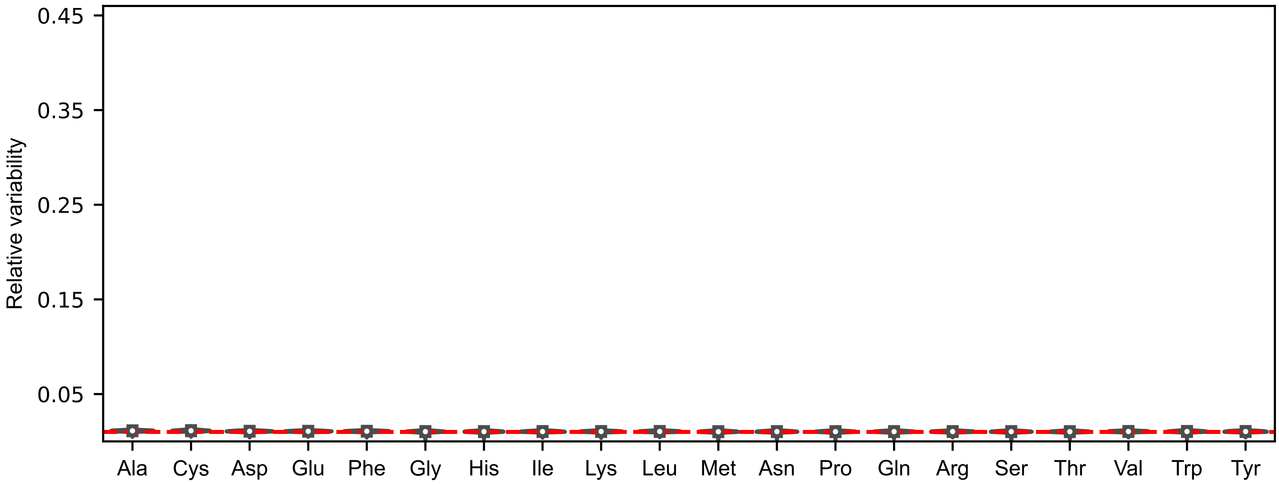

Figure S2. Violin plots of the distributions of mean-normalized flux ranges across $N = 5000$ sampled nutrient combinations for the 20 proteinogenic amino acids of the acidFBA-GEM using an invariant amino acid distribution for all GECKO-implemented proteins. The feasible flux ranges were simulated by performing a flux variability analysis (FVA) using an optimality threshold of 99%. Dotted line in red denote the selected deviation from growth optimality.
